# Perceptions, attitudes, and current practices regards delirium in China: A survey of 917 critical care nurses and physicians in China

**DOI:** 10.1097/MD.0000000000008028

**Published:** 2017-09-29

**Authors:** Jinyan Xing, Yunbo Sun, Yaqi Jie, Zhiyong Yuan, Wenjuan Liu

**Affiliations:** a Department of Critical Care Medicine, The Affiliated Hospital of Qingdao University; b Qingdao Development Zone No.1 Middle School; c School of Nursing, Qingdao University, Qingdao, China.

**Keywords:** critical care, delirium, healthcare professionals, intensive care unit, mechanical ventilation, sedatives, survey

## Abstract

The purpose of this study is to assess the knowledge, attitudes, and managements regarding delirium of intensive care nurses and physicans, and to assess the perceived barriers related to intensive care unit (ICU) delirium monitoring in China. A descriptive survey was distributed to 1156 critical care nurses and physicians from 74 tertiary and secondary hospitals across Shandong province, China. The overall response rate was 86.18% (n = 917). The majority of respondents (88%) believed that deirium was associated with prolonged mechanical ventilation, and 79.72% thought delirium was associated with prolonged length of hospitalization. Only 14.17% of respondents believed that delirium was common in the ICU setting. Only 25.62% of the respondents reported routine screening of ICU delirium, and only 15.81% utilized Confusion Assessment Method for Intensive Care Unit screening tools. “Lack of appropriate screening tools” and “time restraints” were the most common perceived barriers. 45.4% of the participants had never received any education on ICU delirium. In conclusion, most nurses and physicians consider ICU delirium to be a serious problem, but lack knowledge on delirium and monitor this condition poorly. The survey infers a disconnection between the perceived significance and current monitoring of ICU delirium. There is a critical unmet need for in-service education on ICU delirium for physicians and nurses in China.

## 1 Introduction

Delirium is a common disorder in the intensive care unit (ICU) and has been considered as an independent predictor of mortality and morbidity for critically ill patients.^[1]^ ICU delirium is an acute brain dysfunction characterized by alteration in mental status in combination with disorganized thinking and inattention. It consists of 3 subtypes including hyeractive, hypoactive, and mixed delirium.^[2]^ Among the 3 subtypes, mixed and hypoacitive ICU delirium are more common subtypes, accounting for 53% and 36%, respectively; while the hyperactive delirium is relatively less common (11%).^[2]^ Delirium affects up to 80% criticallly ill patients in the ICU setting, but has been under recognized by clinical staff globally.^[1,3–8]^

Daily screening for ICU delirium has been recommended by the Intensive Care Society.^[1]^ Delirium screening tools such as the Intensive Care Delirium Screening Checklist (ICDSC) and the Confusion Assessment Method for Intensive Care Unit (CAM-ICU) have been recommended to be most valid and reliable for routinely delirium monitoring in adult ICU patients.^[1]^ It has been reported that up to two thirds of ICU delirium cases might be missed without a reliable screening tool.^[9]^ However, dispite the high incidence rate and serious negative impacts of delirium, as well as the efforts in implementing delirium scrrening programs, delirium screening is still far less completed by critical care nurses or physicians as reported in werstern countries.^[3,10–12]^

China is the most populous country worldwide, with a large number of medical institutions and ICU centers. However, to the best of our knowledge, only 2 small-scale of surveies regarding ICU delirium have been reported, one of which only included 2 ICUs of Zhejiang University hosipitals^[5]^ and the other only surveyed the anesthetists during an anesthesia forum.^[7]^ Collectively, their results reported an significant disparity between the percieved importance of ICU delirium and current practice.^[5,7]^ Till now, large-scale systematic investigation of ICU healthcare professionals on ICU delirium has been rather limited in China. It is well understood that the successfully widespread implimentation of assessment tools for measuring delirium relies on the medical community's beliefs, perceptions, and attitudes toward delirium. Therefore, in the present study, we carried out a large-scale survey involving critical care physicians and nurses regarding the current knowledge, attitude, and practices of delirium, as well as the perceived barriers to delirium monitoring.

## 2 Methods

### 2.1 Design, setting, and sample

A descriptive survey designed with self-report questionnaire was employed. During the period from February to March 2016, the survey was printed out and distributed to 1156 critical care nurses and physicians at a ratio of 2:1 by investigators from an independent third party. The participants were from all tertiary and most secondary hospitals (74 hospitals in total) located in the 17 cities across Shandong province, China. The study was approved by the Affiliated Hospital of Qingdao University Ethics Committee, and informed consent was assumed with the participation of the survey.

### 2.2 Qestionnare constuction

The questionnare in the present study was constructed by panels of specialists based on a wide review of literature^[3,13–16]^ and clinical practice guidelines.^[1]^ The questionnare mainly consisted of 4 sections: demographics, perception of ICU delirium, attitudes toward ICU delirium, and current practices associated with delirium screening in the ICU setting. Considering the ease and efficiency of collecting responses,^[17]^ the questionnare mainly consists of close response questions with a predetemined list of response options. Many questions allowed for multiple responses.

### 2.3 Pilot work

The validity and reliability of the constructed questionnaire were firstly tested by distribution to 30 ICU delirium experts including 10 ICU physicians and 20 nurses. All experts commented on the clarity and relevance of each survey item. The overall Cronbach α of the questionnaire was 0.814, suggesting good consistency reliability. The question was further adapted and refined according to the feedback from the pilot.

### 2.4 Data collection

All respondents were voluntarily participating in the investigation and could discontinue the survey at any time. The survey was anonymous. The objectives and procedures were explained to the participants in detail before the implementing the survey. Informed consent was assumed with the paticipatation of the survey. All quesionnares were independently filled out by the participants and collected on the spot by invesitgaters from a third party. Once the data was processed and the study completed, the questionnair was destroyed to maintain confidentiality and anonymity.

### 2.5 Data analysis

Data from the survey were recorded by SPSS (version 21.0, Chicago, IL). The data were reported as percentages and frequencies.

## 3 Results

### 3.1 Demographics

In total, 1156 questionnaires were disseminated in 74 hospitals located in all main cities in Shandong province in China. A total of 1064 questionnaries were collected. Around 147 questionnaires were excluded for missing answers (>10%) and repeatable answers. Thus, the overall response rate was 86.18% (n = 917). The median age of participants was 31 years, ranging from 18 to 55 years. The majority of participants (76.23%) were from class A tertiray hospitals (Table 1).

**Table 1:**
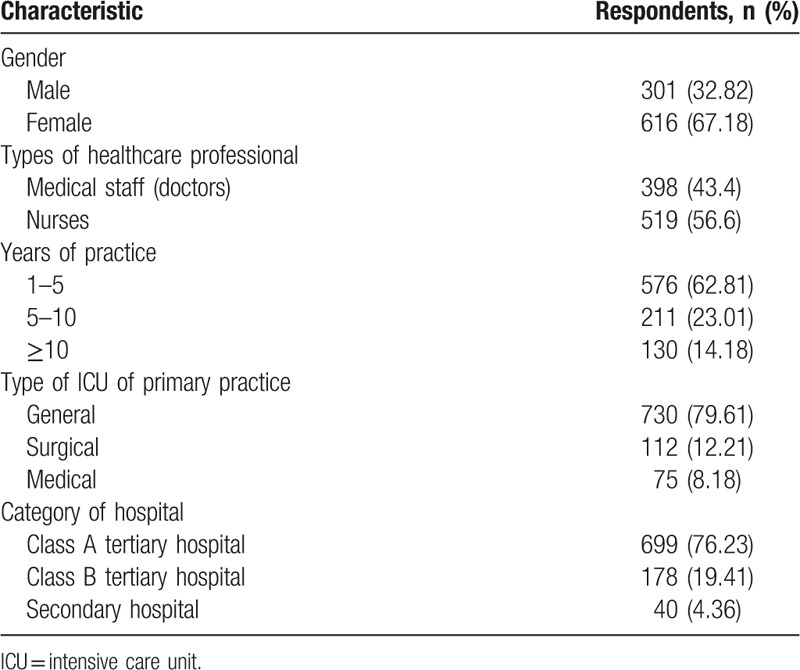
Demographic characteristics of respondents.

### 3.2 Basic knowledge of ICU delirium

Based on the self-report perception of ICU delirium, the survey revealed a high-level knowledge on delirium. Above 90% of particpants stated that they were clear about the definition, classification, and clinical symptoms of delirium. However, when asked about the main symptoms of ICU delirium, 23.8% (n = 218) of respondants believed that “cognitive disorder” was the only main clinical symtom. Similarly, 51.3% (n = 470) of participants took hyeractive delirium as the most common type, while only 12.4% of participants believed hypoactive delirium was the most prevalent type. In addition, only 36.53% (n = 335) of respondents thought they were familiar with screening tools of ICU delirium.

In regard to the perceived prevalence of delirium, almost half of the paticipants (44.27%, n = 406) believed that delirium was rare or had less occurrence in the ICU setting. Only 14.17% of respondents believed that delirium was common in ICU settings. More than half (59.98, n = 550) of the respondents regarded delirium as a normal part of ICU hospitalization (Table 2).

**Table 2:**
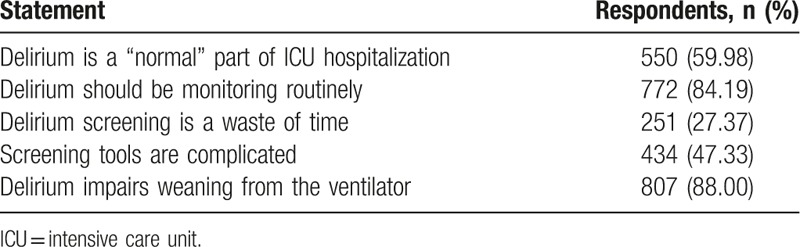
Attitudes on selected issues toward ICU delirium.

### 3.3 Outcomes of delirium

The majority of respondents (88%, n = 807) believed that deirium was associated with prolonged mechanical ventilation (Table 2). 79.72% (n = 731) of respondents thought delirium was associated with prolonged length of stay in hospital. In terms of motality, 77.54% (n = 711) agreed that ICU delirium was in association with higher motality for critically ill patients.

### 3.4 Current practices regarding ICU delirium aseessment

The data indicated that only 25.62% (n = 235) of the medical stuff reported routine screening of delirium in the ICU. Nearly half (41.77%, n = 383) of the participants assessed ICU delirium but not routinely. In addition, 17.78% (n = 163) claimed that they carried out sedation evaluation, indicating a confusion of evaluation of delirium and sedation (Table 3).

**Table 3:**
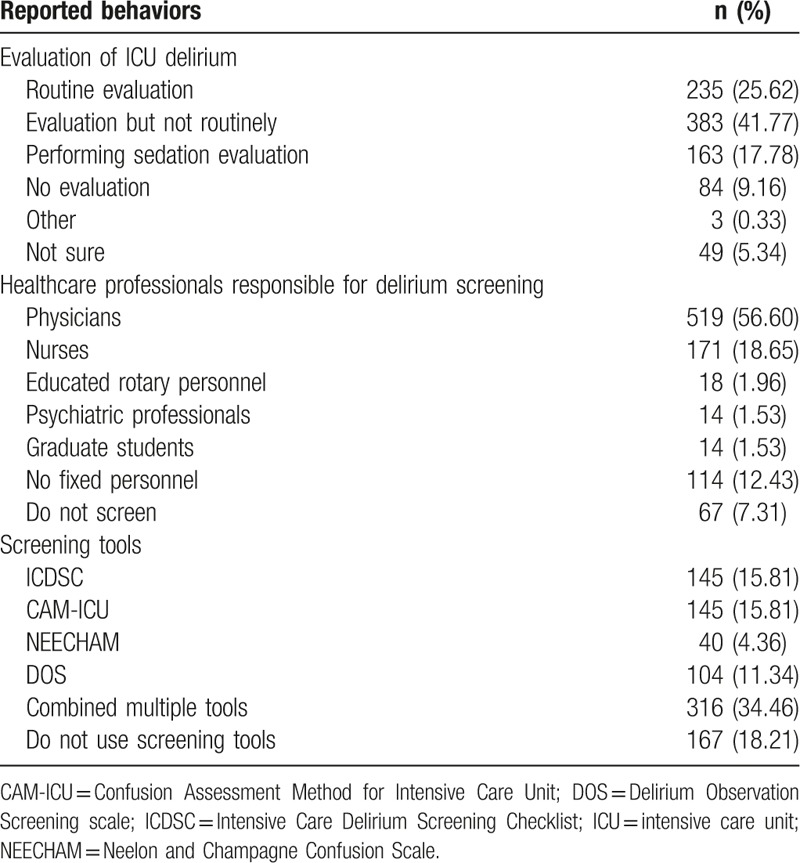
Reported behaviors regarding delirium screening.

When respondents were asked, “who are responsible for delirium screening?,” the most frequent response was physicians (56.60%, n = 519), followed by nurses (18.65%, n = 171) and no fixed personnels (12.43%, n = 114) (Table 3).

Ony 19.41% (n = 178) of participants reported the initial evaluation of delirium was performed within 24 hours after admission, while almost half of the respondents (49.18%, n = 451) selected the response “it depends.” When asking about the frequency of delirium screening, 23.56% (n = 216) selected “on a daily basis”; 11.01% (n = 101) selected “once per shift”; and almost half (47.55%, n = 436) selected “it depends.”

Despite the poor mangement of ICU delirium, the data revealed a high percentage (81.79%) of respondents using specific assessment tools for screening ICU delirium, with only 18.21% (n = 167) of respondents reported that they did not use screening tools. However, a closer look at the data showed that only 15.81% (n = 145) of respondents utilized ICDSC for delirium screening, and another 15.81% (n = 145) utilized CAM-ICU. It should be noted that 34.46% of respondents (n = 316) stated that multiple screening tools were applied for delirium monitoring. This is virtually impossible considering the complexity of screening tools and medical staff deficiency caused by the relative huge number of patients in China.

### 3.5 Education on delirium

The data indicated that 45.4% (n = 416) of the participants had never received any education or training on ICU delirium previously. More than 3 quarters of respondents (87.7%, n = 804) stated a desire to receive relevant training on delirium.

### 3.6 Barriers to ICU delirium screening

Although the majority (84.19%, n = 772) of the participants believed that routine delirium monitoring should be implemented in the ICU setting (Table 2), only 25.62% (n = 235) reported routine screening of delirium. When asked about the “percieved barriers to ICU delirium screening,” the most frequent response was “lack of appropriate screening tools” (49.18%, n = 451), followed closely by other 3 responses, including “heavy workload caused lack of comuniciation with paitients” (48.53%, n = 445), “time consuming for applying delirium screening tools to detect delirium” (47.33%, n = 434), and “insufficient knowledge of ICU delirium” (46.01%, n = 422) (Table 4).

**Table 4:**
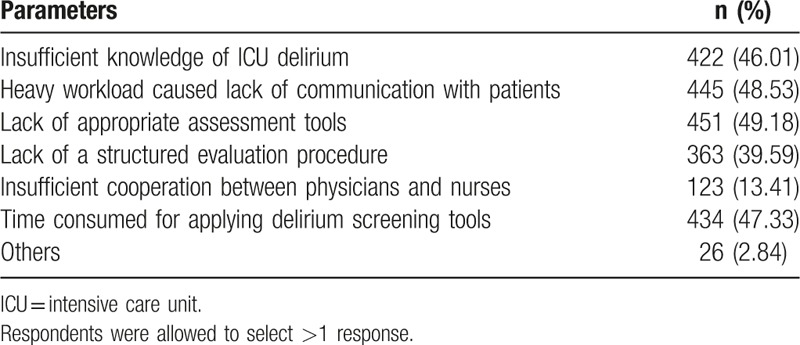
Barriers to screening for delirium in the ICU.

## 4 Discussion

This report represents the first large-scale survey of the perception, attitudes, current practices, and perceived barriers regarding ICU delirium of physicians and nurses in China. The survey revealed that >90% of the respondents stated that they had a high level of knowledge regarding delirium. However, a large portion of them even did not clear about the main clinical symptoms of delirium. This suggests that although the respondents are informed of ICU delirium, they may actually lack of detailed knowledge in its symptoms, associated complications, and subsequent assessment tools of ICU delirium. Similar results have been obtained regarding the most common type of delirium in this survey. Over half of respondents thought hyeractive delirium as the most prevalent form. In consistency with previous studies, hypoactive delirium was least recognized type of ICU delirium, whereas hyperactive delirium was more commonly considered to be the most prevalent subtype.^[18]^ The survey data also revealed that physicians and nurses believed that delirium in critically ill patients was a serious problem and was associated with prolonged mechanical ventilation and higher motality. As reported by multiple studies, the incidence rate of delirium is 20% to 80% in the ICU setting.^[1,3,8,11,19–26]^ Chen et al^[27]^ reported an incidence rate of 25.8% of delirium in their ICU in China. Similarily, Su et al^[28]^ found that incidence of postoperative delirium was 23% in patients after noncardiac surgery. The incidence of delirium in our department is approximately 25% to 30% based on our daily assessments. However, almost half (44.27%) of participants believed that delirium was rare or less occurrence in the ICU setting in this survey. The underestimated incidence of delirium may be associated with a lack of knowledge on delirium. The reported low level of education on delirium may be a possible reason for the inadequate knowledge. Taken together, this survey suggests that there is a critical unmet need for in-service education on delirium for physicians and nurses in the ICU setting.

Routine delirium screening is the premise of early detection and timely treatment in the ICU setting. The opinions of medical staff regarding the importance of ICU delirium monitoring were not reflected in their practices, according to the data of the survey. Although the majority (84.19%) of the participants believed that routine delirium monitoring should be implemented in the ICU setting, only 25.62% reported routine screening of delirium. In consistency with previous studies, our results revealed an obvious disparity between the perceived significance of delirium and current practices toward ICU delirium.^[5,12,29,30]^ In addition, 17.78% of respondents reported that they carried out sedation evaluation. Considering that sedation evaluation is only a part of delirium screening,^[28]^ the results indicate that a nonnegligible proportion of participants may be confused about delirium and sedation assessment, further suggesting an insufficient knowledge on delirium assessments.

CAM-ICU and ICDSC were the most commonly used assessment tools with great reliability and validity.^[1,27,28]^ This survey revealed that ∼16% of participants reported the use of CAM-ICU, and another 16% reported the use of ICDSC. In contrast, 34.46% of the respondents stated that they monitored delirium with multiple assessment tools. From the complexity of the current evaluation tools and our clinical practice experience, the data may not reflect the actual evaluation status. These participants may have a great likelihood of not implementing screening tools at all. Regarding the 18.21% of respondents who reported no use of screening tools, they may not perform delirium assessment with great likelihood. The low rate of utilizing screening instruments may be due to a lack of relative domestic clinical guidelines for ICU delirium in China. Studies revealed that only less than half of healthcare professionals used proper assessment tools in delirium monitoring in western counties, even though there have been extensive studies and great effort in implementing delirium screening.^[29,30]^ Our results suggest that it is in urgent need to develop support structures for the frontline medical stuff in China. The healthcare management team is responsible to develop domestic and preformulated clinical guidelines and provide in-service education routinely. It is obvious that, in addition to the negative impacts of delirium on the patients, failing to detect and treat delirium will increase national healthcare budgets. This can be minimized through developing domestic and preformulated clinical guidelines, as well as poviding regular in-service education and training nationally.

Despite the clinical guidelines and literature that persisted emphasizing the importance for delirium screening and the necessity of using specific screening tools,^[1,31,32]^ “lack of appropriate screening tools” was still the most frequently reported barrier to delirium monitoring in this study. This may be caused by the lack of domestic clinical guidelines in China. Besides, time restraints have also been reported to be a frequently associated barrier to ICU delirium monitoring.^[30]^ Patel et al^[3]^ reported that ICU delirium screening was considered to take up valuable time from nurses and medical staff. Similarly, we have found that almost half of the respondents believed that it is time consuming for applying relative screening tools to detect delirium.

The survey had several limitations. Similar with many surveys, self-reporting responses may be accompanied with inevitable inaccuracies due to response bias which could have caused by poor recollection of clinical experiences or misunderstanding of questions. Because the questionnaires were distributed and collected *via* paper and pen on the spot, the response rate was greater than mailing. However, this may interfere with the in-depth thinking of participants which may further expand the response bias. In addition, this survey only included ICU centers in Shandong Province from China, which may affect the generalizability of the study. However, Shandong province is the China's second populous province with ∼95 million inhabitants at 2010 Census and is the third wealthiest province with a GDP of US$967 billion in 2014. Besides, our survey covered all tertiary hospitals and most secondary hospitals across Shandong province. Therefore, our results may importantly reflect the general status of delirium monitoring in China.

In conclusion, most nurses and physicians consider that delirium is a serious problem, but lack knowledge on delirium and monitor this condition poorly. The survey infers a disconnection between the perceived significance and current monitoring of ICU delirium. There is a critical unmet need for in-service education and training on ICU delirium for critical care physicians and nurses in China.
